# Laparoscopic lateral duodenojejunostomy for pediatric superior mesenteric artery compression syndrome: a cohort retrospective study

**DOI:** 10.1186/s12893-023-02274-2

**Published:** 2023-12-04

**Authors:** Jingfeng Tang, Mengxin Zhang, Ying Zhou, Guoqing Cao, Shuai Li, Xi Zhang, Shaotao Tang

**Affiliations:** 1 Department of Hepatobiliary Surgery, Union Hospital, Tongji Medical College, Huazhong University of Science and Technology, Wuhan, China; 2 Department of Pediatric Surgery, Union Hospital, Tongji Medical College, Huazhong University of Science and Technology, Wuhan, China

**Keywords:** Children, Laparoscopic surgery, Superior mesenteric artery syndrome, Duodenojejunostomy

## Abstract

**Purpose:**

There are only a few case reports of laparoscopic lateral duodenojejunostomy (LLDJ) in children with Wilkie’s syndrome, also known as superior mesenteric artery compression syndrome (SMAS). We aimed to describe our laparoscopic technique and evaluate its outcomes for SMAS in children.

**Methods:**

From January 2013 to May 2021, SMAS children who received LLDJ were included. The procedure was carried out utilizing the four-trocar technique. The elevation of the transverse colon allows good exposure of the dilated and bulging second and third sections of the duodenum. Using a linear stapler, we established a lateral anastomosis connecting the proximal jejunum with the third part of the duodenum. Following that, a running suture was used to intracorporeally close the common enterotomy. Clinical data on patients was collected for analysis. The demographics, diagnostic findings, and postoperative outcomes were analyzed retrospectively.

**Results:**

We retrospectively analyzed 9 SMAS patients (6 females and 3 males) who underwent LLDJ, aged between 7 and 17 years old. The mean operative time was 118.4 ± 16.5 min and the mean estimated blood loss was 5.6 ± 1.4 ml. There were no conversion, intraoperative complications or immediate postoperative complications. The mean postoperative hospital stay was 6.8 ± 1.9 days and the mean follow-up time was 5.4 ± 3.0 years. During follow-up, seven patients (77.8%) experienced complete recovery of symptoms prior to surgery. One patient (11.1%) still had mild vomiting, which resolved with medication. Another patient (11.1%) developed psychological-induced nausea, which significantly improved after treatment with education, training and diet management.

**Conclusions:**

LLDJ represents a feasible and safe treatment option for SMAS in well-selected children. Further evaluation with more cases and case-control studies is required for the real benefits.

## Introduction

Superior mesenteric artery syndrome (SMAS), an uncommon disorder, compresses the third part of the duodenum and has a mortality rate as high as 33% [1]. Mechanical obstruction resulting from compression of the third section of the duodenum, deriving anterior from the superior mesenteric artery (SMA) and posterior from the aorta and vertebral column, is the widely accepted definition [2]. Since SMAS was first described in 1862 [3], the pathogenesis has been deeply investigated and further understood. Children with developmental delay may have a higher incidence than adults (0.013–0.300%) [2, 4], and surgical approaches are similar in children and adults. Thanks to the rapid development of laparoscopic techniques recently, the laparoscopic approach has become the preferred option for adults [5]. Laparoscopic lateral duodenojejunostomy (LLDJ) has been practiced in children with SMAS in only a few case reports, but its efficacy remains unknown. Thus, we present the results of 9 pediatric SMAS patients treated with LLDJ and share technical details. This is the largest series of SMAS children treated with LLDJ to date.

## Patients and methods

### Patients

**Fig. 1:**
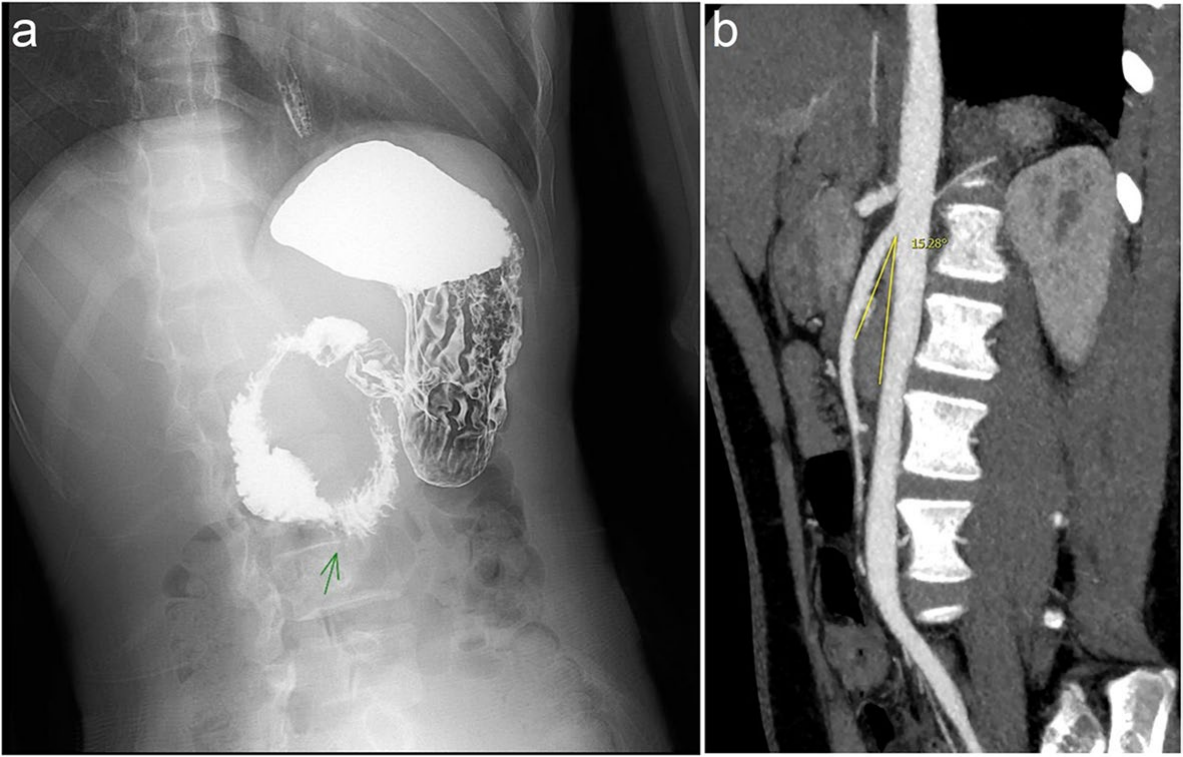
Image data of 8 years old girl with SMAS. **a** Barium meal picture “penholder sign” and **b** The aortomesenteric angle

Nine children with SMAS treated by LLDJ in our department between January 2013 to May 2021 were reviewed retrospectively. The procedures were carried out by same surgical team. This study was approved by the Ethics Committee at our institution. Informed consents were obtained from parents of all study participants preoperatively. Nine patients with suspected SMAS were referred after confirmation by radiographic studies (barium meal, CT scan or ultrasonography). Their findings included proximal duodenal dilation with the “penholder sign” in the 3rd portion in association with an angle formed by the SMA (Fig. 1a) and the aota of < 25° consistent with SMAS (Fig. 1b). The initial treatment of all patients was treated conservatively including medication (antacids, histamine H_2_ receptor antagonist, proton pump inhibitors, or prokinetics), postural therapy, and parenteral nutrition. Among them, 2 children underwent enteral feeding through a nasojejunal tube, and 1 child received anti-depressant therapy. When patient’s symptoms did not show improvement following non-operative treatment, a second consultation for surgical evaluation is necessary. Indicators included demographics, surgical outcome and further clinical treatment. All patients had good compliance and were able to cooperate well with doctors to receive treatment.

### Operative techniques

**Fig. 2:**
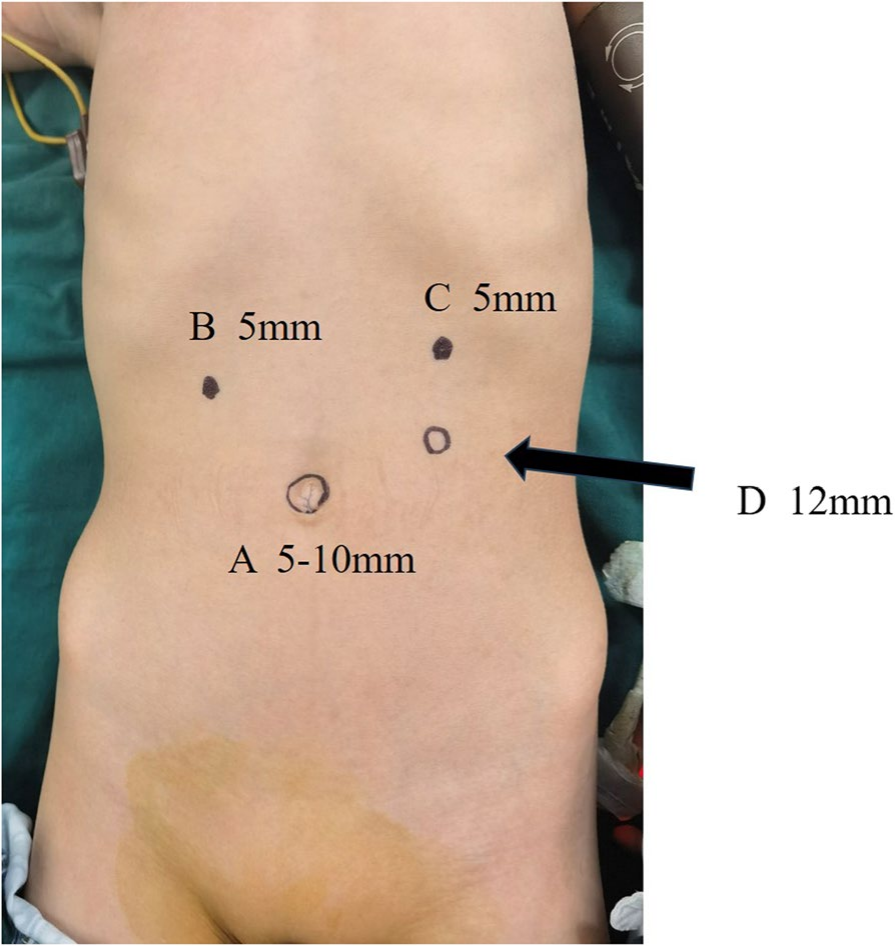
Trocar placement

**Fig. 3:**
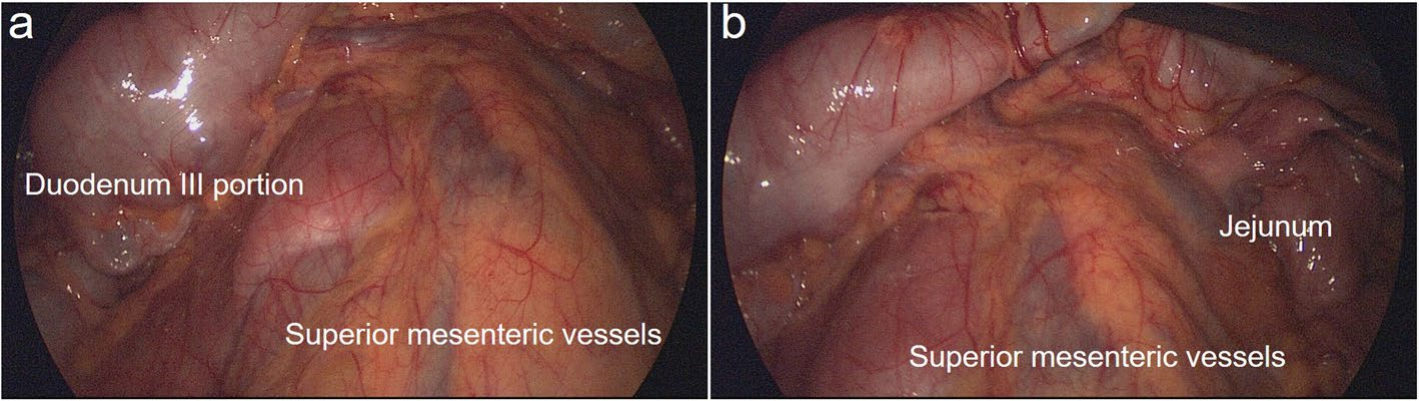
Third portion of the duodenum, the superior mesenteric artery and the beginning of the jejunum

Placing the patient in a reverse Trendelenburg position (20°-30°) and administering general anesthesia, we inserted a 5- or 10-mm trocar (A) through the umbilicus to accommodate the scope while setting a carbon dioxide pneumoperitoneum pressure of 8–10 mmHg. As depicted in Fig. 2, we placed two 5-mm trocars between the umbilicus and costal margin, on the right (B) and left (C) midclavicular lines. A 12-mm trocar (D) was placed between the umbilicus and “C” port in the left lumbar area. The dilated second and third parts of the duodenum (adjacent to the superior mesenteric artery) were exposed by elevating the transverse colon and omentum (Fig. 3). We utilized a hook cautery to cut the covering visceral peritoneum, freeing this part of the duodenum from the retroperitoneum. The jejunum loop (10–15 cm distal to the ligament of Treitz) was identified and then moved beneath the third segment of the duodenum.

**Fig. 4:**
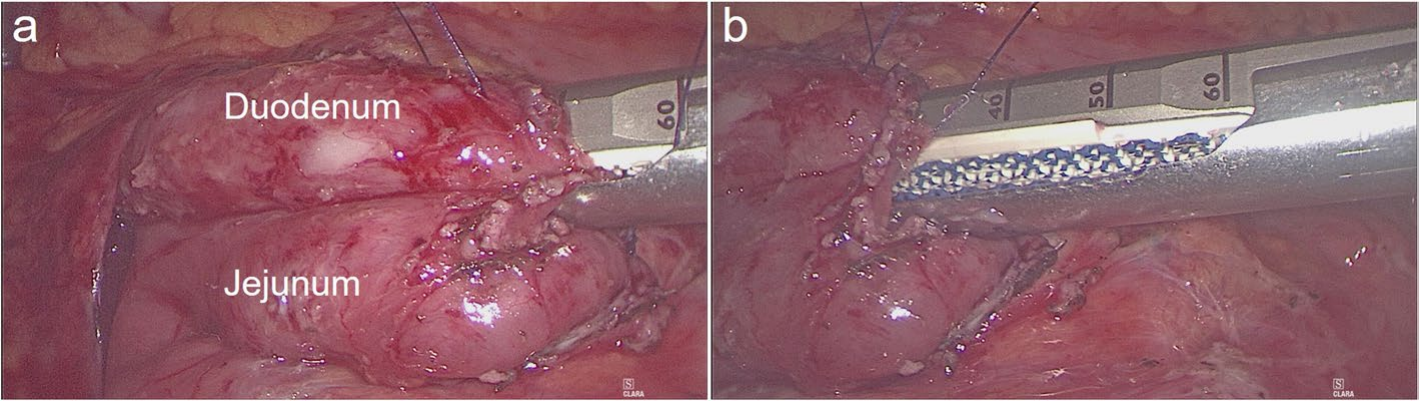
The duodenojejunostomy was performed as a side-to-side anastomosis

**Fig. 5:**
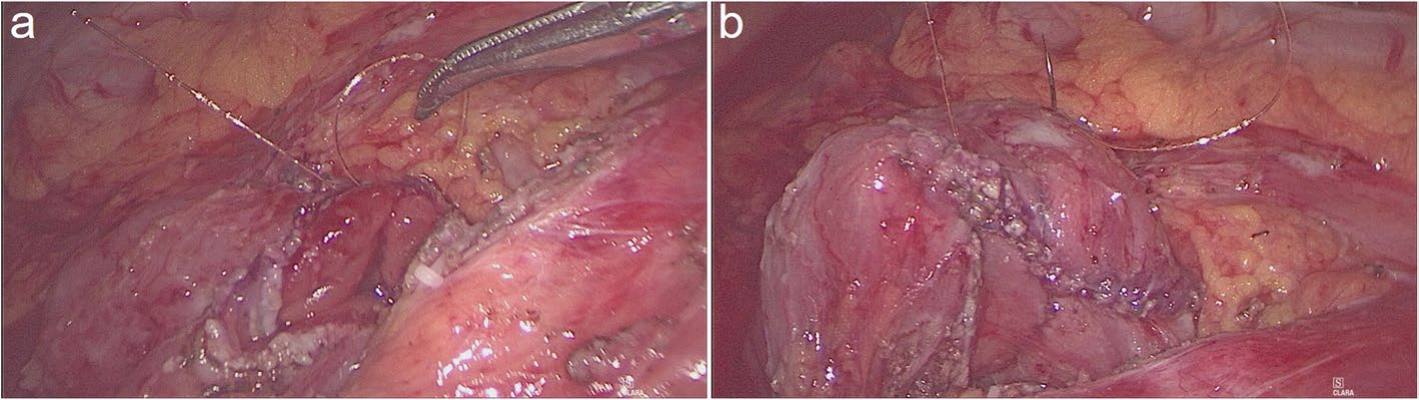
The common enterotomy is closed intracorporeally with a 4–0 unidirectional barbed running suture (in a single layer)

We used 4–0 absorbable sutures as stay sutures, followed by minor openings of approximately 1 cm created in both the jejunum and duodenum. The two jaws of a 60 mm linear stapler were introduced into the jejunal and duodenal lumens, respectively. The duodenojejunostomy was performed as a side-to-side anastomosis (Fig. 4). Subsequently, the enterotomy was closed intracorporeally in a single layer using a 4–0 unidirectional barbed running suture (Fig. 5), and the intermesenteric gap was sealed through the application of interrupted silk sutures. An abdominal drainage tube was not required. The postoperative diet of patients was gradually transitioned from a liquid diet to a regular diet.

After discharge, patients were followed routinely in the outpatient clinic (at postoperative 1, 3, and 6 months and every 6 months thereafter). At each outpatient visit, an experienced physician would evaluate the patient’s BMI and symptoms, such as nausea, vomiting, abdominal distension, epigastric pain, and so on. Moreover, blood routine and radiographic examinations (barium meal and ultrasonography) were performed if necessary.

### Statistical analysis

Descriptive statistical analysis of the variables in this study was conducted using SPSS version 26.0. The mean ± standard deviation (SD) or median (range) was used to represent continuous variables, while the number (n) and percentages (%) were used to show categorical variables.

## Results

A total of 9 patients (6 females and 3 males) with SMAS who underwent LLDJ were enrolled in our study. These patients’ diagnoses were based on clinical symptoms and imaging findings, including upper gastrointestinal barium studies in 2 patients (obstruction in the third portion with dilation in the first and second portions of the duodenum), CT angiography in 4 patients (a narrowed aortomesenteric angle), and both tests in 3 patients (Fig. 1). The median age at surgery was 12.3 years (range: 7–17 years), and no conversion or complications occurred during the operation. The mean operative time was 118.4 ± 16.5 min, accompanied by minimal blood loss (5.6 ± 1.4 ml). The mean hospital stay length was 6.8 ± 1.9 days and the mean follow-up time was 5.4 ± 3.0 years. There were no wound infections or anastomotic complications in this study. The clinical symptoms of obstruction were significantly improved or eliminated in 7 patients (77.8%). During follow-up, one patient (11.1%) experienced greater relief from nausea and generalized epigastric pain, yet the vomiting still existed, which was relieved after 3 months of antiacid and spasmolysis treatment. One patient (11.1%) encountered psychological-induced nausea, which was significantly alleviated by eating solid food. She had significant improvement after 5 months of treatment with education, training and diet management. Following surgery, imaging demonstrated favorable emptying of both the stomach and duodenum, with no signs of recurring obstruction. Notably, all patients’ nutritional status exhibited marked improvement.

## Discussion

SMAS commonly occurs in females and tends to affect young people aged 10–39 years [6, 7], with a median age of 23 years reported in the literature [8]. The youngest case reported so far is an infant aged 6 months [9]. Management of SMAS patients often begins with conservative treatment, encompassing gastrointestinal decompression and parenteral nutrition totally to alleviate obstruction while preventing dehydration and maintaining electrolyte balance. While a universally accepted standard treatment strategy for SMAS in the pediatric population has yet to be established, conservative treatment is usually preferred as the initial approach. However, nonoperative efforts cannot solve the root problem. Once conservative treatment is ineffective, surgical intervention is necessary for symptomatic patients. A minimum of 6 weeks of medical therapy prior to surgical intervention has been recommended [10], but the optimal duration remains unclear. It’s important to recognize that requiring a child to retain a nasojejunal tube for several weeks to complete conservative treatment can be quite challenging. Nonetheless, if medical treatment proves ineffective or continuous vomiting results in additional weight loss, surgical correction becomes necessary [11]. Moreover, based on two comparable cohorts of children in 1974 and 2006, the need for surgical intervention in pediatric SMAS surged from 14 to 70% [12].

Currently, surgical treatments for pediatric SMAS include gastrojejunostomy, lysis of the Treitz ligament, Ladd’s procedure, Roux-en-Y duodenojejunostomy and lateral duodenojejunostomy. Gastrojejunostomy serves as a treatment option, offering effective gastric decompression of duodenal obstruction; however, it carries the risk of complications, including incomplete relief of duodenal obstruction, peptic ulceration, and blind loop syndrome, all of which can contribute to the ongoing presence of symptoms [13]. Its application is limited due to poor results in the treatment of chronic SMAS [14]. It is suggested that gastrojejunostomy is a feasible remedy for patients with severe abdominal adhesions that may make duodenojejunostomy impossible [15]. A Strong’s technique (lysis of the Treitz ligament and division of the fourth part of the duodenum) permits the duodenum to be mobilized caudally outside the aortomesenteric angle, but its drawback is a 25% failure rate resulting from tethering of the inferior pancreaticoduodenal artery, even if appealing because bowel integrity is not compromised by an anastomosis. In addition, it may form a narrow mesenteric appendage with a lifelong risk of volvulus, especially in children [16, 17]. The Ladd’s procedure, as an anastomose-free technique, allows for the preservation of intact bowel flow [9]. Although this technique is easy to perform, its efficacy needs to be further confirmed. Currently, the most widely used surgical procedure for SMAS is duodenojejunostomy. Some studies have reported that lateral duodenojejunostomy (LDJ) is superior to the above procedures based on higher success rates and lower potential complications [18–20]. Transabdominal LDJ dates to 1908 and remains the standard therapy for SMAS [21].

Laparoscopic techniques have been spread over the last 30 years and are gradually being applied to SMAS. Gersin et al. [22] performed the first LLDJ for an adult in 1998, who obtained a good postoperative recovery and was discharged without complications. Although no prospective trials are available, many of the open procedures could be performed with minimally invasive methods, producing similar or better outcomes. In 2006, Palanivelu [14] carried out the first successful LLDJ procedure in a child aged 14 years old, with a postoperative contrast study confirming unobstructed flow to the jejunum. Since then, more and more LLDJ have been performed successfully in pediatric SMAS cases. Following a review of the literature, we have identified 18 cases (Table 1 [9, 14–16, 23–33]) of laparoscopic techniques employed in children (< 18 years): 12 LLDJ cases, 4 laparoscopic Ladd’s procedure cases, 2 laparoscopic Roux-en-Y duodenojejunostomy cases and 1 laparoscopic duodenum lysis case. These procedures have the benefits of rapid recovery, functional improvement in bowel motility and the patient’s health, decreased chances of small bowel adhesions and postoperative incisional hernia, minimum blood loss, less pain postoperatively, and good cosmetic outcomes.

**Table 1:** Case reports of laparoscopic surgery for pediatric patients with SMAS

Author	Year	Gender	Age	Treatment	Operative time (min)	Conversion	Follow-up (months)	Postoperative complications	Recurrence
B Li [9]	2020	Female	6 months	Ladd’s procedure	65	None	34	None	None
B Li [9]	2020	Male	9 months	Ladd’s procedure	75	None	16	None	None
B Li [9]	2020	Female	9 months	Ladd’s procedure	60	None	12	None	None
C Palanivelu [14]	2006	Male	14 yeras	LLDJ	110	None	6	None	None
PS Cullis [23]	2016	Female	12 yeras	LLDJ	200	None	5	Small volumes of diet within 2 weeks, followed by an improvement in appetite over the next 4 months	None
R Kumar [24]	2016	Male	15 yeras	LLDJ	140	None	6	None	None
GC Kirby [15]	2017	Male	17 yeras	LLDJ	NA	None	9.3	None	None
L Barkhatov [25]	2018	Female	14 yeras	LLDJ	95	None	≥ 12	None	None
RR Rai [26]	2019	Female	13 yeras	LLDJ	NA	None	6	None	None
JL Record [27]	2015	Female	13 yeras	LLDJ	NA	None	12	Self-limited upper gastrointestinal bleed (no need for transfusion)	None
JL Record [27]	2015	Female	16 yeras	LLDJ	NA	None	0.5	None	None
Castro B Aneiros [28]	2018	Male	13 yeras	LLDJ	125	None	13	None	None
Castro B Aneiros [28]	2018	Male	13 yeras	LLDJ	75	None	13	None	None
QH Wang [29]	2010	Female	17 yeras	Laparoscopic lysis of duodenum	NA	None	12	None	None
A Kurbegov [30]	2010	Female	16 yeras	LLDJ	NA	None	NA	None	None
M Alsulaimy [31]	2014	Female	17 yeras	Ladd’s procedure	NA	None	NA	None	None
H Muhammad [32]	2022	Male	16 yeras	LLDJ	NA	None	NA	None	None
M Sato [16]	2015	Female	6 yeras	Roux-en-Y	217	None	9	None	None
Li J [33]	2011	Female	17 yeras	Roux-en-Y	110	None	24	Intermittent vomiting within 3 months postoperatively and no symptoms thereafter	None

NA: not clear

Although laparoscopic Roux-en-Y duodenojejunal is regarded as a more physiological reconstruction to prevent reverse antiperistalsis, it does heighten technical complexity and time consumption [34]. Based on this, we are more interested in LLDJ, which is easy and feasible, achieving good decompression of the third portion of the duodenum and having functional bypass. LLDJ is relatively easier to perform than the laparoscopic Roux-en-Y procedure, especially for children [31]. We hold the opinion that the reverse peristalsis can be removed with the large size of the anastomosis. We used a 60-mm suture cutter in operations, which usually apply to adults. As shown in our case series, all nine patients successfully achieved significant relief of SMAS by LLDJ, and patients did not develop worsening vomiting or symptoms of reverse persistence after surgery. Previous studies have focused on the safety of LLDJ and found that it is safe in children with SMAS, with significant relief from symptoms, no recurrence, and no serious postoperative complications, although only individual cases have been reported (Table 1) [14, 15, 23–28, 30]. These findings support our results, and we have evaluated more pediatric cases to provide further evidence to confirm the feasibility and effectiveness of LLDJ. We believe that LLDJ is expected to become an appropriate choice for SMAS in children, which has the inherent advantages of easy operation, high technical feasibility and good efficacy. Interestingly, one special case with perioperative complications was identified. The patient continued to have nausea and vomiting, but interestingly, imaging findings postoperatively revealed positive gastric and duodenal emptying with no further evidence of obstruction. We consider that the patient might have a preoperative psychological condition such as anorexia or a partial eclipse that was caused or concealed by the symptoms of the SMAS. After training and diet management, the patient reported symptom improvement. The connection between anorexia nervosa, drug abuse, and other eating disorders is well-documented in previous studies [35, 36]. Therefore, it is essential that surgical management ensure optimal long-term outcomes in collaboration with psychologists and dieticians. Among adults who underwent laparoscopic duodenojejunostomy, only 33.3% of patients obtained improvement in symptoms during the intermediate follow-up. Whereas, 77.8% (7/9) of the children in our series had improved or resolved symptoms. This may be due to the fact that postoperative psychological status has less influence on the clinical symptoms of SMA in children compared to adults.

We admit that there are limitations to our study. The retrospective design, small sample size and non-comparative study are major limitations. However, the strength of our inclusion of nine patients is the largest case analysis of pediatric SMAS treated surgically under the same criteria since it is a relatively rare disease and our case series obtained satisfactory results. The advantages and risks of various available procedures should be fully considered in light of each patient’s specific conditions. Further clinical trials are needed to verify our findings.

In conclusion, LLDJ is a feasible and safe treatment option for SMAS in well-selected children, with easy operation, high technical feasibility and satisfactory outcomes. Further evaluation is required to identify the real benefits.

## Data Availability

The datasets used and/or analysed during the current study are available from the corresponding author on reasonable request.

## Abbreviations

SMAS: Superior mesenteric artery compression syndrome
LLDJ: Laparoscopic lateral duodenojejunostomy
SMA: Superior mesenteric artery
